# Current knowledge of the species complex *Anastrepha fraterculus* (Diptera, Tephritidae) in Brazil

**DOI:** 10.3897/zookeys.540.9791

**Published:** 2015-11-26

**Authors:** Lucie Vaníčková, Vicente Hernández-Ortiz, Iara Sordi Joachim Bravo, Vanessa Dias, Alzira Kelly Passos Roriz, Raul Alberto Laumann, Adriana de Lima Mendonça, Beatriz Aguiar Jordão Paranhos, Ruth Rufino do Nascimento

**Affiliations:** 1 Laboratório de Ecologia Química, Instituto de Química e Biotecnologia, Universidade Federal de Alagoas, Av. Lourival de Melo Mota, s/n, Tabuleiro, 57072-970, Maceió, AL, Brazil; 2 Instituto de Ecología A.C., Red de Interacciones Multitróficas. Carretera Antigua a Coatepec 351, El Haya, 91070, Xalapa, Veracruz, Mexico; 3 Universidade Federal da Bahia, Instituto de Biologia, Departamento da Biologia Geral. R. Barão do Geremoabo s/n, Campus Universitário de Ondina, 40170-290, Salvador, BA, Brazil; 4 University of Florida, Gainesville, FL 32611, United States; 5 Empresa Brasileira de Pesquisa Agropecuária (EMBRAPA), Centro Nacional de Pesquisa de Recursos Genéticos e Biotecnologia, Parque Estação Biológica W5 Norte / Final Asa Norte, 70770917, Brasília, DF, Brazil; 6 Empresa Brasileira de Pesquisa Agropecuária (EMBRAPA-Semiárido), BR 428, Zona Rural, 56302-970, Petrolina, PE, Brazil

**Keywords:** South American fruit fly, cryptic species, taxonomy, sexual behavior, chemical communication, acoustic communication

## Abstract

The study of the species complex *Anastrepha fraterculus* (*Af* complex) in Brazil is especially important in a taxonomical, evolutionary and pest management context, because there are evidences that some of them may occur in sympatry. In this review, we analyzed the main results supporting evidences that three cryptic species occur in Brazil. The taxonomical and phylogenetic relationships based on eggshell morphology, adult morphometrics, as well as cytotaxonomy and genetic differentiations are discussed. We also review available information on sexual behavior including acoustic communication of males during courtship and sexual incompatibility; and chemical signals involved in the communication between sexes, with a special focus on sex pheromones. We examined the role of long- and short-range pheromones (male-produced volatiles and cuticular hydrocarbons, respectively), their implications in sexual isolation, and their possible use for chemotaxonomic differentiation of the putative species of the *Af* complex.

## Introduction

The fruit fly *Anastrepha fraterculus* constitutes a complex of cryptic species (*Af* complex) currently composed of eight taxonomically recognized morphotypes (Hernández-Ortiz et al. 2012, 2015). Its geographical distribution ranges from Southern Texas through Eastern Mexico, Central and South America (Stone 1942, Hernández-Ortiz and Aluja 1993, Norrbom et al. 1999). In Brazil, *Anastrepha fraterculus* is one of the most important polyphagous pests infesting about 70 host plant species (Zucchi 2007, 2008). Several studies confirmed that natural Brazilian populations of *Anastrepha fraterculus* have morphological, biological, and genetic differences throughout their geographical distribution (Stone 1942, Malavasi and Morgante 1983, Steck 1991, Selivon and Perondini 1998, Selivon et al. 1997, 1999, 2004, 2005a,b, Silva and Barr 2008). Three entities of the *Af* complex, termed as *Anastrepha* sp.1 aff. *fraterculus*, *Anastrepha* sp.2 aff. *fraterculus*, and *Anastrepha* sp.3 aff. *fraterculus*, occur in Brazil (Yamada and Selivon 2001, Selivon et al. 2004, 2005a). Although, an extensive review of genetics and biology of *Anastrepha fraterculus* from Argentina has been published (Cladera et al. 2014), information about the ecology, taxonomy, and behavior of *Anastrepha fraterculus* putative species in Brazil is still insufficient and this imposes constrains to implementation of environmental friendly control methods, such as the Sterile Insect Technique (SIT) (Dyck et al. 2005). In order to apply SIT, insect strains are reared on a massive scale in facilities with the potential to produce millions of sterile insects per week (Hendrichs et al. 1995). Compatibility between wild and laboratory reared insects is critical for the success of this pest management method (Wong et al. 1982, Cayol 1999, Meza-Hernández and Díaz-Fleischer 2006, Benelli et al. 2014a).

Here, we present a revised synthesis on the current status of our knowledge of the *Anastrepha fraterculus* complex in Brazil, focusing on divergence among Brazilian populations by evaluating multiple aspects: (i) taxonomy and relationships, (ii) sexual behavior and reproductive incompatibility, and (iii) chemical communication between sexes.

## Taxonomy and relationships

The first documented evidence of a cryptic species complex appeared in the comprehensive taxonomic revision of the genus *Anastrepha* by Stone (1942). He described extensive morphological variation among specimens from Mexico through South America and considered these samples to constitute geographical races. Stone stated: “As treated here it [*Anastrepha fraterculus*] extends from the Rio Grande valley in Texas south to Argentina, and it is possible that it will eventually be found to represent a complex of species rather than a single one.”

Since then, enough information has been gathered to affirm that the nominal species *Anastrepha fraterculus* in fact represents a cryptic species complex (*Af* complex). Some studies conducted in the 1990’s correlated morphological traits and genetics of Brazilian samples (Selivon and Perondini 1998, Selivon et al. 1996, 1997, 1999), enabling the recognition of two different biological entities within the complex (referred in this paper as *Anastrepha* sp.1 or Brazilian-1 and *Anastrepha* sp.2 or Brazilian-2). Later Selivon et al. (2004, 2005a,b) used diverse sources and proposed the existence of a third Brazilian entity named *Anastrepha* sp.3 aff. *fraterculus* (abbreviated in this paper as *Anastrepha* sp.3 or Brazilian-3).

### Adult morphology

**Figure 1.**
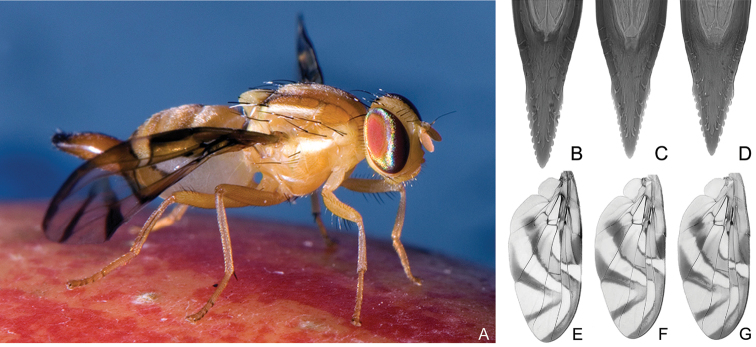
Adult female *Anastrepha fraterculus* (**A**) and typical forms of the aculeus tip and the wing pattern of morphotypes Brazilian-1 (**B, E**), Brazilian-2 (**C, F**), Brazilian-3 (**D, G**), respectively. (The photo of adult was made by Dr. Hoskovec, the images of aculeus and wings were modified from Hernández-Ortiz et al. 2012).

The historical taxonomy of the genus *Anastrepha* is largely based on adult characters of the female aculeus, external morphology of the body, and the wing pattern (Stone 1942, Steyskal 1977) (Figure 1). Although males of many species currently cannot be distinguished at all, recent studies have found characters in the male genitalia useful for identification of some species groups and phylogenetic relationships (Norrbom et al. 1999, 2012).

Taxonomic studies of the nominal *Anastrepha fraterculus* (*sensu lato*) done by Lutz and Lima (1918), Lima (1934), Greene (1934) and later by Stone (1942), showed high variability among adult populations, so currently six synonyms are based on Brazilian specimens: *Tephritis mellea* Walker, 1837 (St. Paul’s, Brazil); Anastrepha fraterculus var. soluta Bezzii, 1909 (São Paulo, Brazil); *Anastrepha braziliensis* Greene, 1934 (Viçosa, Minas Gerais, Brazil); *Anastrepha costarukmanii* Capoor, 1954 (Itajuba, Minas Gerais, Brazil); *Anastrepha scholae* Capoor, 1955 (Agua Preta, Bahia, Brazil); *Anastrepha pseudofraterculus* Capoor, 1955 (Itatiaia, Rio de Janeiro, Brazil).

Besides, morphometric assessment proved to be useful for the recognition of *Anastrepha* species. Araujo and Zucchi (2006) performed a discriminant function analysis on linear measures of the aculeus to separate Brazilian samples of *Anastrepha fraterculus* (*s.l.*) from *Anastrepha obliqua* (Macquart), *Anastrepha sororcula* Zucchi, *Anastrepha zenildae* Zucchi and *Anastrepha turpiniae* Stone. Recently geometric morphometrics of the wing has been also used for the recognition of species such as *Anastrepha fraterculus* (*s. l.*), *Anastrepha obliqua*, and *Anastrepha sororcula* (Perre et al. 2014). All species are related as they were classified within the *“fraterculus* species group” (*sensu* Norrbom et al. 2012). Comparative studies on morphometrics of the wing of males and females, and the aculeus tip was done with Brazilian *Anastrepha* sp.1 and *Anastrepha* sp.2. These results demonstrated that wing morphometrics could be used to distinguish these species efficiently, and wing sexual dimorphism was also recognized (Selivon et al. 2005a).

However, based on adult morphology, the first evidence of differences among Brazilian populations of the *Af* complex from other countries was made with comparisons of seven samples from Mexico, two from Brazil (São Paulo and Piracicaba), and each one from Argentina (Tucumán) and Colombia (Tolima) (Hernández-Ortiz et al. 2004). They used measures of the aculeus, wing and mesonotum of females. These data clearly separated a Mexican morphotype, and the Brazilian and the Argentinean samples were clearly differentiated from the Colombian sample, resulting in the naming of these clusters as Brazilian and Andean morphotypes, respectively.

Further morphometric analyses using 32 populations from Mexico, Central America, and South America (including Venezuela, Colombia, Ecuador, Peru, Brazil and Argentina), confirmed previous findings and clearly added that within the *Af* complex seven morphotypes could be discerned throughout the Neotropical region (Hernández-Ortiz et al. 2012). The eight Brazilian populations examined belong to the biogeographical sub-regions Chacoan and Paranaense, distinguishing three discrete clusters nominated morphotypes; the Brazilian-1 morphotype comprised samples from the states of São Paulo, Santa Catarina, and Minas Gerais; the Brazilian-2 morphotype was represented by two samples from Ilha Bela and São Sebastião (state of São Paulo); and the Brazilian-3 morphotype was characterized by a single sample from Ubatuba (São Paulo).

### Egg morphology

**Figure 2.**
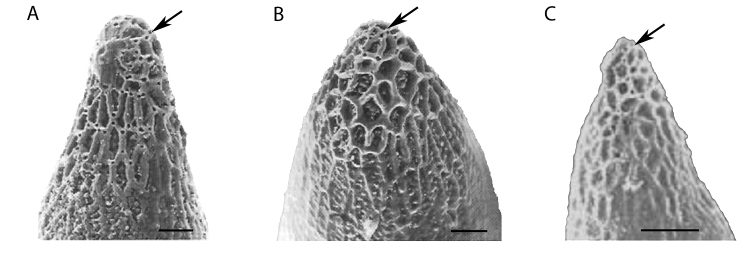
Scanning electron micrographs of the anterior tip (dorsal view) of eggs from Brazilian-1 (**A**), Brazilian-2 (**B**), Brazilian-3 (**C**) morphotype of *Anastrepha fraterculus*. The arrow shows aeropyles. Bars = 20 µm (**A, B**) and 50 µm (**C**). The images were modified from Selivon et al. 2004, and from Selivon and Perondini 1998, with permision.

Differences in egg morphology discovered between Brazilian populations of the *Af* complex suggested the existence of two different taxonomic entities for the first time. Through scanning electron microscopy, Selivon and Perondini (1998) described the external morphology of the chorion characterizing two distinct biological entities called *Anastrepha fraterculus* sp.1 and *Anastrepha fraterculus* sp.2, which were later named *Anastrepha* sp.1 aff. *fraterculus* and *Anastrepha* sp.2 aff. *fraterculus* (Yamada and Selivon 2001). Eggs of *Anastrepha* sp.1 are smaller than those of *Anastrepha* sp.2; in the former there is a papilla at the anterior pole which is absent in the *Anastrepha* sp.2; the micropyle is closer to the apex in *Anastrepha* sp.2; the anterior pole is ornamented by folds of the chorion forming irregular polygons in both, although in eggs of *Anastrepha* sp.2 these folds are arranged in a rosette around the micropyle. Aeropyles are found almost exclusively on the ventral side of the anterior pole, being more numerous in *Anastrepha* sp.1. Later Selivon et al. (2004) described the eggs of Brazilian *Anastrepha* sp.3, showing that it differs from *Anastrepha* sp.1 and *Anastrepha* sp.2 in terms of size, position of the micropyle, and the ornamentation of the chorion at the anterior pole (Figure 2).

### Geographic distribution and host use

**Figure 3.**
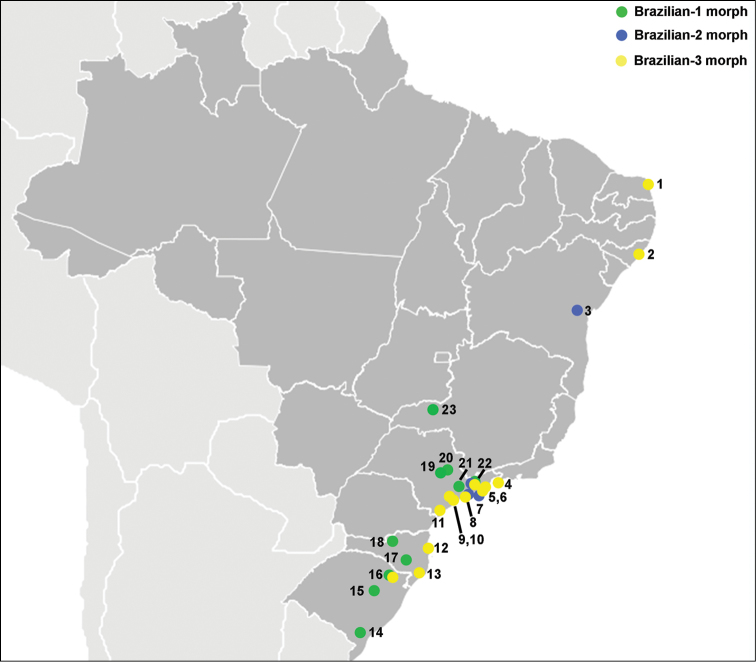
Map of Brazil indicating the geographic locations from which Brazilian-1 (green), Brazilian-2 (blue) and Brazilian-3 (yellow) morphotypes of *Anastrepha fraterculus* were collected. **1** Parnamirim, RN **2** Maceio, AL **3** Conceição de Almeida, BA **4** Parati, RJ **5** Ubatuba, SP **6** Caraguatatuba, SP **7** Ilhabela, SP **8** São Sebastião, SP (region of sympatry of Brazilian-2 and Brazilian-3 morphotypes, Selivon et al. 2004, Hernández-Ortiz et al. 2012) **9** Maresias, SP **10** Miracatu, SP **11** Morretes, SP **12** Porto Belo, SC **13** Criciúma, SC **14** Pelotas, RS **15** Bento Gonçalves, RS **16** Vacaria, RS (region of sympatry of Brazilian-1 and Brazilian-3 morphotypes, Selivon et al. 2004) **17** São Joaquim, SC **18** Caçador, SC **19** Botucatu, SP **20** Piracicaba, SP **21** São Paulo, SP **22** Santa Isabel and Jambeiro, SP (Paraíba valley - region of sympatry of the three morphotypes, Selivon et al. 2004) **23** Uberlandia, MG.

Concerning the species distribution and host use of the Brazilian members of the complex, there is very limited information and few inferences can be made. For example, two species of the Brazilian complex, *Anastrepha* sp.1 and *Anastrepha* sp.2 exhibited preferentially an allopatric distributional pattern. However, in most of 18 locations sampled from Brazilian Inland Plateau, they can be found together infesting guavas (*Psidium guajava*) and oranges (*Citrus* sp.), respectively, and only two locations in the Paraíba valley (Santa Isabel and Jambeiro, in the state of São Paulo) recorded the co-occurrence of the three Brazilian forms (Selivon et al. 2004, Selivon and Perondini 2007). Other species, *Anastrepha* sp.3 was very common in the Atlantic coastal region (in the states of Rio de Janeiro, São Paulo, and Santa Catarina), and no records were documented in most locations from Brazilian Inland Plateau (in the states of Santa Catarina, Paraná, São Paulo, Goiás, and Minas Gerais) (Figure 3). Therefore, *Anastrepha* sp.3 seems to be restricted to the coastal plain areas, where it co-occurs with *Anastrepha* sp.2 and can be found even infesting the same host fruits, guava or tropical almond (*Terminalia catappa*). Food preferences of the *Af* complex remain uncertain, since about 70 host plant species have been recorded in Brazil for the nominal *Anastrepha fraterculus* (*s.l.*) (Zucchi 2007, 2008). While a number of these records may be questionable due to possible misidentification of fruit flies, understanding the relationships of the Brazilian entities with their native hosts will be of great relevance to determine the pest status of each taxonomic entity within the species complex.

### Cytotaxonomy

The first cytological evidence of differences between samples of the *Af* complex was reported by Bush (1962). He compared the karyotypes of a Mexican sample respective to the Brazilian population described by Mendes (1958). Bush suggested that this difference might represent a case of chromosomal polymorphism or, more likely, sibling species. This conclusion was supported based on morphological differences attributed to geographical variation and distinct host preferences because the Brazilian population is a pest of citrus, while the Mexican population does not infest citrus and therefore is not considered economically important in Mexico.

Later Solferini and Morgante (1987) studied the karyotype of eight *Anastrepha* species from Brazil, highlighting that all of them could be identified on the basis of chromosome morphology. Samples from six Brazilian localities were studied – Itaquera, Sorocaba, São Roque (from São Paulo state); Conceição do Almeida, Cruz das Almas, and Santo Antonio de Jesus (from Bahia state). Four distinguishable karyotypes were found; two of them from the state of São Paulo, and two others from the state of Bahia, suggesting that they represent sibling species. However, one karyotype described from Bahia actually corresponded to *Anastrepha sororcula* Zucchi (Morgante et al. 1993). In the nominal species *Anastrepha fraterculus*, similar acrocentric autosomes and chromosome numbers (2n=12) have been described from populations in Mexico, Brazil and Argentina (Mendes 1958, Bush 1962, Solferini and Morgante 1987, Selivon et al. 1996, Selivon et al. 1997, Basso et al. 2003). In the Brazilian species complex, conspicuous differences in the sex chromosomes were found. Chromosomes X and Y were larger in *Anastrepha* sp.2 respective to *Anastrepha* sp.1 and *Anastrepha* sp.3; in addition, to differences in the distribution and location of the blocks of constitutive heterochromatin (Selivon et al. 2004, 2005a).

More recently, Goday et al. (2006) performed a comparative analysis of heterochromatin organization in the sex chromosomes to determine the rDNA loci. They used sequential staining techniques with DAPI and chromomycin A3 fluorochromes, which have different affinities for DNA bases, followed by C-banding. A specific sex–chromosome banding pattern was obtained. This technique demonstrated structural differences on the Y chromosome between *Anastrepha* sp.1 and *Anastrepha* sp.3, allowing an accurate separation of these two species with this method.

### Genetics

The first molecular study of intraspecific variation in the *Af* complex in Brazil was performed by Morgante et al. (1980) using an isozyme electrophoresis analysis. They studied 11 enzymatic loci for 16 populations of *Anastrepha fraterculus* (*s. l.*) coming from six localities of southern, southeastern and northeastern Brazil [Itaquera, Sorocaba, Sao Roque (São Paulo); Conceição do Almeida, Cruz das Almas, and Santo Antonio de Jesus (Bahia)]. They summarized the *“Af* complex” as consisting of four population subgroups, with the northeastern populations being more different from the others. However, one member of the subgroup from northeastern Brazil (Bahia) was later determined to belong to *Anastrepha sororcula* Zucchi (Morgante et al. 1993), and corresponding to the karyotype 3 formerly described by Solferini and Morgante (1987).

A similar isozymic analysis conducted by Steck (1991) involved samples spanning a wide geographical range (Mexico, Costa Rica, Venezuela, Peru and Brazil). His results showed strong genetic differentiation within the nominal *Anastrepha fraterculus*. Extreme frequency and/or fixed allele differences were found among samples from Andean vs lowland Venezuela and also between Brazilian samples from the south (São Paulo) vs the northeast (Bahia). Separation among samples were far greater than any observed among populations of the reference species, suggesting that the *Af* complex as it now stands may even not be monophyletic (Steck 1999). Shortly thereafter, Steck and Sheppard (1993) corroborated the findings of isozyme data by using mitochondrial DNA restriction fragment length polymorphism (RFLP). This method separated populations from northeast and southeast Brazil. They also demonstrated that specimens from coastal Venezuela and from the Bahia region were highly differentiated, even though they originally seemed similar based on isozymic analyses.

Studies of mitochondrial DNA (Santos 1994) also reported strong evidence of large inter-population variability in Brazilian samples, recognizing the existence of two haplotypes within the nominal species *Anastrepha fraterculus* when compared with *Anastrepha obliqua* and *Anastrepha sororcula*. McPheron et al. (1999) investigated 16S rDNA data to analyze the relationships among *Anastrepha* species, however they only included two sequences of the *Af* complex. They found that a single specimen from Mérida (Venezuela) was distinct from a specimen collected in the state of São Paulo (Brazil). The phylogenetic relationships inferred from mtDNA sequences of COI by Smith-Caldas et al. (2001), further supported the presence of multiple gene pools within the nominal *Anastrepha fraterculus*. They suggested a cryptic species exists in the high elevations of the Andes and further corroborated the non-monophyly among samples of the *Af* complex.

Rocha and Selivon (2004) analyzed samples involving the three Brazilian entities, subjecting the total DNA to fragmentation by restriction endonucleases. The banding pattern showed specificity among species with *Anastrepha* sp.3 being very distinct from *Anastrepha* sp.1 and *Anastrepha* sp.2. Some bands were common to species of the *Af* complex and six other *Anastrepha* species as well. However, other bands were only observed in the three entities of the *Af* complex but in different arrangements within the genome (Selivon and Perondini 2007). Selivon et al. (2005a) conducted combined analyses of isozymes, karyotypes, morphometry, and crossings from 10 Brazilian populations. The isozymic study comprised a survey of 16 enzymatic systems of 19 loci, and results showed significant differences in the allele frequencies at four loci (FUM, ME, HEX, and LDH). Results showed the presence of two clearly distinct genetic clusters, which were related differentially with other species of the *fraterculus* species group, suggesting that *Af* complex would not be monophyletic. The most important finding of this work was that both clusters differed in the length of their sex chromosomes and the size and location of heterochromatic regions.

Barr et al. (2005) used the nuclear gene *period* to reconstruct the phylogeny of *Anastrepha*, but their tree included only seven specimens of *Anastrepha fraterculus*, four from Venezuela (Mérida and Caracas), two from México, and a single one from Brazil (São Paulo). The Brazilian specimen was distinct from the two other clades composed by Mexican and Venezuelan specimens, further suggesting that the nominal *Anastrepha fraterculus* is not monophyletic.

Vaníčková (2012) performed a comparative study of COII and ITS1 using two populations of *Anastrepha fraterculus* (Bento Gonçalves, Rio Grande do Sul; Tucumán, Argentina). The sequenced parts of the COII gene were not different. The sequencing of ITS1 gene resulted in AT-rich sequences (84%) and released tandem-repeats/poly-N stretches and poly-A-stretches. Nevertheless, the variability of the sequences was very low. These results confirmed that the studied populations belong to the same Brazilian-1 morphotype. Further studies including brother populations sampling were suggested in order to confirm COII and ITS1 as suitable genes for resolution of the three Brazilian forms inside the *Af* complex.

Recently, Silva et al. (2014) investigated the variability of COI among 200 specimens of *Anastrepha fraterculus* sampled from Brazil, Mexico, and Argentina. The COI genetic variation in *Anastrepha fraterculus* was high. Three haplotypes were exclusive to Brazilian collections, one to Argentina, and five to Mexico. For Brazil, the most common haplotype was seen among 73% of the samples. These authors concluded that based on the phylogenies and geography of samples, the COI gene has limited utility in recognizing cryptic species.

Unfortunately, available data as a whole do not permit correlating different karyotypes or genetic and molecular traits, with the morphology and distribution of the Brazilian sibling species, mainly because studies were carried out with flies from different locations. A synthesis of molecular datasets from the existing literature is precluded because the original authors applied different methodologies or genetic loci to analyse *Anastrepha fraterculus* samples (Silva and Barr 2008).

## Sexual behavior and reproductive incompatibility

The lek polygyny mating system displayed by *Anastrepha fraterculus* was first described by Malavasi et al. (1983) through systematic field observation. Calling males aggregate in the top of host and non-host trees from the first hour after dawn until mid-morning, forming groups in which males fight to defend a small territory where they court females and mate (Segura et al. 2007, Benelli et al. 2014b). In *Anastrepha fraterculus* male aggressions are not frequent and/or outcomes are not crucial for mating success (Segura et al. 2007, Benelli 2015a, 2015b). The courtship behavior exhibited by *Anastrepha fraterculus* lekking males is complex and composed of visual, acoustical, and chemical displays (Mankin et al. 1996, Segura et al. 2007, Gomez Cendra et al. 2011). Differences in either time of mating or patterns of courtship behavior among species from the *Af* complex have the potential to affect mating recognition and ultimately lead to reproductive isolation (Morgante et al. 1983, Selivon et al. 1999, Vera et al. 2006, Cáceres et al. 2009, Segura et al. 2011, Dias 2012, Rull et al. 2013, Devescovi et al. 2014). Herein, time of mating, patterns of courtship behavior, aspects of reproductive compatibility/incompatibility and acoustic communication will be reviewed regarding the Brazilian populations of the *Af* complex.

### Courtship behavior

Overall, time of mating and the period in which *Af* complex males from the studied Brazilian populations display their courtship behavior in leks seem to occur mainly in the morning, shortly after the sunrise (Malavasi et al. 1983, Lima et al. 2001, Vera et al. 2006, Rull et al. 2012, Dias 2012). So far, differences in mate timing have not been reported yet for Brazilian entities of the *Af* complex.

Lek formation and courtship displays were compared among five Brazilian populations of the *Af* complex from South (Bento Gonçalves, Pelotas, and Vacaria – Rio Grande do Sul; São Joaquim – Santa Catarina) and Southeast regions of Brazil (Piracicaba – São Paulo) by Dias (2012). Males from both Brazilian regions seem to be attracted to the same leks because no differences were found in the male lek distribution in field cages; however, males from the same regions differed in the frequency of some courtship displays. According to Dias (2012), 12 behavioral units, defined as distinct steps of male courtship, characterize the sexual behavior displayed by *Af* complex males from South and Southeast Brazil and comparisons made on five behavioral unit frequencies associated with mating success revealed differences among them.

### Reproductive incompatibility

Although Brazil is the South American country with the highest number of *Af* complex entities (Hernández-Ortiz et al. 2012), little is known about the sexual compatibility of its *Anastrepha fraterculus* populations. Partial postzygotic isolation between two Brazilian populations, determined as *Anastrepha* sp.1 and *Anastrepha* sp.2, was reported by Selivon et al. (1999). In this work, some degree of postzygotic reproductive isolation was found among F1 crosses between *Anastrepha* sp.1 males (Vacaria, Rio Grande do Sul) and *Anastrepha* sp.2 females (Conceição do Almeida, Bahia). Cytoplasmic incompatibility between different *Wolbachia* strains found in eggs of *Anastrepha* sp.1 and *Anastrepha* sp.2 has been suggested as one of the causes of their postzygotic reproductive isolation (Selivon et al. 1996; Selivon et al. 1999; Selivon et al. 2002). Later, *Wolbachia* strains were also found in adults from Piracicaba (Cáceres et al. 2009) as well as in *Anastrepha* sp. 1, *Anastrepha* sp.2, and *Anastrepha* sp. 3 from Southeastern Brazil (Marcon et al. 2011). Postzygotic reproductive isolation was reported by Selivon et al. (2005a) based on crosses between two population clusters of *Af* complex from Northeastern (Rio Grande do Norte, Bahia), Southeastern (Minas Gerais, São Paulo), and Southern (Rio Grande do Sul) Brazil, corroborating a previous study (Selivon et al. 1999).

Vera et al. (2006) found partial sexual isolation between *Anastrepha fraterculus* from Piracicaba (São Paulo) and Argentina (Tucumán), as well as high sexual isolation between them and two Peruvian (La Molina, Piura) *Anastrepha fraterculus* populations. Dias (2012) reported full sexual compatibility among *Anastrepha fraterculus* populations from the south region of Brazil (Bento Gonçalves, Pelotas, Vacaria, and São Joaquim), but partial sexual isolation between flies from the south and southeast (Piracicaba) regions. Rull et al. (2012) found prezygotic and postzygotic reproductive compatibility among three populations of Brazilian-1 morphotype from Pelotas, Vacaria, and Tucumán. In contrast, the same Brazilian populations from Pelotas and Vacaria, both characterized as Brazilian-1 morphotype, showed strong prezygotic isolation when compared to the Mexican morphotype (Xalapa, Veracruz (Rull et al. 2013)). In addition, prezygotic and postzygotic reproductive isolation were found among some populations from the Andean (Ibagué), Mexican (Xalapa), Peruvian (La Molina), Brazilian-1 (Tucumán, Argentina), and Brazilian-3 (Parnamirim, Brazil) morphotypes, patterns potentially due to the presence of *Wolbachia* (Devescovi et al. 2014).

Although some progress has been made toward our understanding about the reproductive isolation barriers among the Brazilian populations of the *Af* complex, this advance is still discrete given the high cryptic species diversity that could be potentially found in the north, northeast, south, and southeast of Brazil. Further studies need to be conducted in order to elucidate the mechanisms involved in the divergence among the cryptic species of the *Af* complex, which could help to predict their distribution.

### Acoustic communication

Acoustic communication during reproductive behavior has been identified in twenty-four species of tephritid flies and characterized in ten species, including some *Anastrepha* spp. (Takata 2010). This type of communication is characteristic of polyphagous Tephritidae in which males form leks (Shelly 2001). In *Anastrepha* species two types of sound have been described. The “calling song” is characterized by rapid backward-forward wing movement that generates pulses trains with pulses variation between 0.1 to 0.5 s duration with interpulses of the same duration and dominant frequency of ca. 80 - 150 Hz. The “precopulatory” song is characterized by continuous wing vibration of about 170 Hz, this song is highly variable in duration lasting few seconds to some minutes (Webb 1983, Sivinski 1984, Mankin 1996, Briceño 2009, Takata 2010).

Intra- and interspecific variations in sound production may be important in the eventual reproductive isolation of species/populations and could contribute to speciation. Mankin et al. (1996) studied differences in song repertoires and characteristics of four species of *Anastrepha* [*Anastrepha fraterculus* (two populations), *Anastrepha obliqua*, *Anastrepha sororcula* and *Anastrepha grandis*] from different regions of Brazil and found that differences in calling sounds produce pre-copulatory reproductive isolation. Although sound is important, pheromone and behavioral differences may play a larger role in separating species (Sivinski and Webb 1985, Sivinski 1988). In the two populations of *Anastrepha fraterculus* [Itaquera (São Paulo), Cruz das Almas (Bahia)] the calling song did not show differences that could contribute to reproductive isolation (Mankin et al. 1996). Similar results were reported by Joachim-Bravo et al. (2013) working with four populations of *Anastrepha fraterculus* from different regions of Brazil, namely Piracicaba (São Paulo), São Joaquim (Santa Catarina), Vacaria (Rio Grande do Sul) and Bento Gonçalves (Rio Grande do Sul). Nevertheless, a recent study that include some of the Brazilian species of the *Af* complex (*Anastrepha* sp.1, *Anastrepha* sp.2, *Anastrepha* sp.3), *Anastrepha obliqua* and *Anastrepha amita* showed the calling song could be a signal used in interspecific recognition contributing to reproductive isolation. The authors concluded that calling songs constitute a relevant factor maintaining the genetic integrity of the species (Takata 2010). Traits that determine reproductive isolation among species are subjected to the stochastic nature of evolutionary forces that might vary across taxa (Lenormand et al. 2009, Nosil et al. 2009). Hence, acoustic communication may be an important factor determining reproductive isolation among *Anastrepha*, but not among taxonomic entities of the *Af* complex. Overall, unless the difference in a particular trait is correlated to female mate preference, sexual isolation among species is questionable (Ritchie 1996, Ritchie et al. 1999).

Future work should test the role that acoustic communication plays on the reproductive behavior and also if the temporal (time components) and spectral (frequency and intensity components) characteristics could be used in species/populations recognition. In addition, vibratory substrate-borne components of acoustic signals could transmit information between individuals; this possibility has not yet been studied in tephritid flies.

## Chemical communication

### Sex pheromones

Sex pheromones play an important role in species/partner recognition and in the mating behavior of *Anastrepha* species (Sivinski 1989, Sugayama and Malavasi 2000). During ‘calling’ (one of the first courtship behaviors), males disseminate a volatile mixture of compounds to attract males and females (Nation 1989, Lima et al. 2001). The studies involving the chemicals which trigger communication in *Anastrepha* species have been conducted considering two distinct perspectives: 1) the way flies store and release their sex pheromones, and 2) how these infochemicals are used to attract females and influence mating (Landolt and Averill 1999). The volatile compounds released by *Anastrepha fraterculus* males were first isolated from salivary gland extracts of specimens from the south of Brazil (Pelotas, Rio Grande do Sul) (Lima et al. 2001). A lactone, (*E*, *E*)-suspensolide, two isomeric sesquiterpenes, (*E*, *Z*)-α-farnesene and (*Z*, *E*)-α-farnesene and four alkylpyrazines, 2,5-dimethylpyrazine, 2,3,5-trimethylpyrazine, 3-ethyl-2,5-dimethylpyrazine, were found in those extracts as major compounds, and 3-butyl-2,5-dimethylpyrazine was detected as a minor component. The monoterpenes, (*Z*)-β-ocimene and limonene, were also identified in the mixture of volatiles released by males, and the isomeric lactones, anastrephin and (*S*, *S*)-epianastrephin, were found in extracts derived from washing the aeration chamber where males were placed for volatile collection (Lima et al. 2001).

A subsequent study carried out by Santos (2003), using *Anastrepha fraterculus* males from northeast Brazilian population (Alagoas), identified 23 compounds in the mixture of volatiles released by the flies. One alcohol, (*E*, *Z*)-3,6-nonadien-1-ol, four sesquiterpenes, α-*trans*-caryophyllene, (*E*, *E*)-α-farnesene, α-*trans*-bergamotene and β-bisabolene and the isomeric lactones, suspensolide, anastrephin and (*S*, *S*)-epianastrephin were reported among the identified compounds.

Further studies on the chemical composition of sexual pheromone of *Anastrepha fraterculus* and its perception by conspecific females were conducted (Břízová et al. 2010, Břízová 2011, Vaníčková 2010, Zyková 2013). The laboratory population used in these studies was *Anastrepha* sp.1 (Tucumán, Argentina) and 5 compounds, which elicited antennal depolarization on conspecific female antenna, were detected from male emanations. These compounds were identified as (*Z*)-3-nonen-1-ol, (*Z*, *Z*)-3,6-nonadien-1-ol, (*E*, *E*)-α-farnesene, (*Z*, *E*)-α-farnesene and epianastrephin. Furthermore, the study on age-dependant changes in the production of male-borne volatiles has shown that quantitative production of volatiles was lower in younger flies (5 days old) and increased with age (15-20 days old) reaching its maximum on the 20th day after eclosion (Zyková 2013).

**Figure 4.**
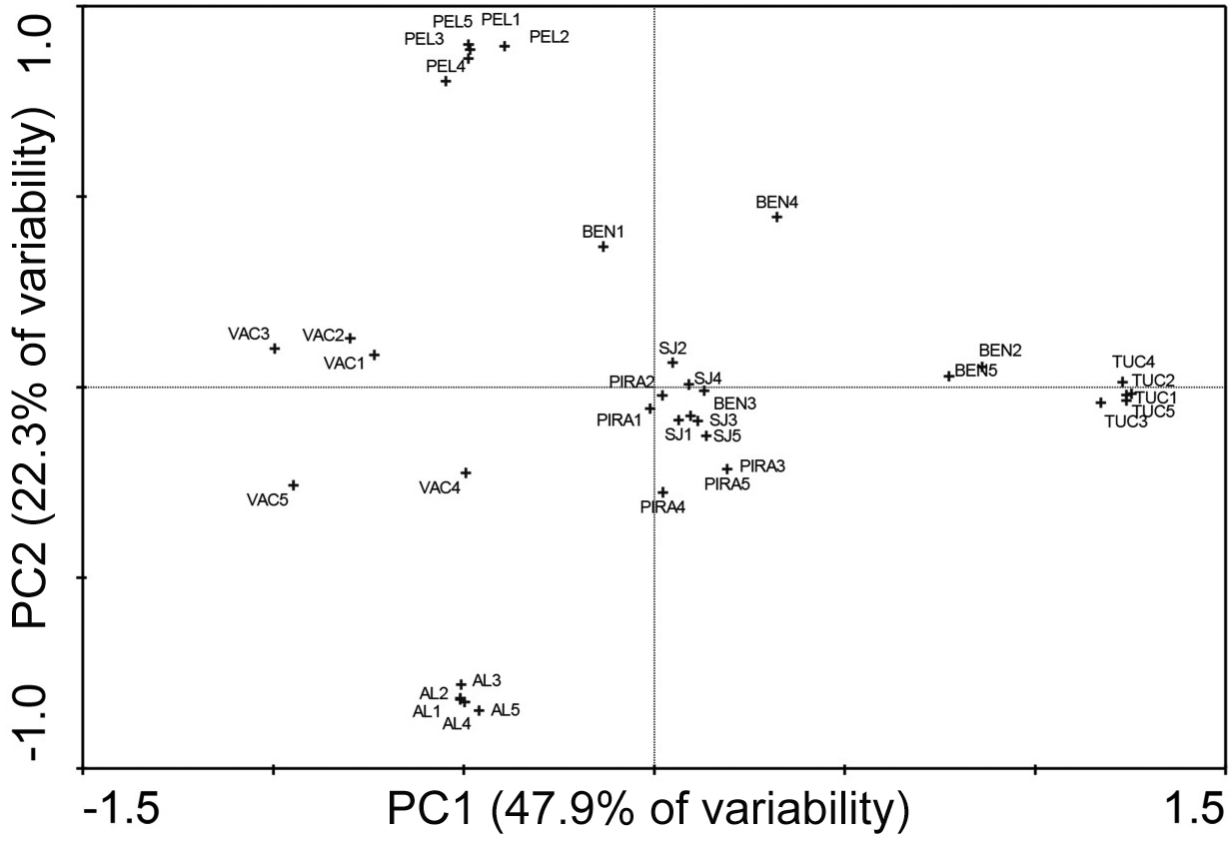
The results of the multivariate principal component analysis (PCA) of the sex pheromone of the males of *Anastrepha fraterculus* from 7 different populations representing two Brazilian morphotypes (A. sp.1, A. sp.3). A. sp.1. PEL – Pelotas (RS, BR), BEN – Bento Gonçalves (RS, BR), VAC – Vacaria (RS, BR), SAO - São Joaquim (SC, BR), TUC – Tucumán (AR); A. sp.3. AL – Alagoas (AL, BR); PIRA – Piracicaba (SP, BR) [modified after Břízová et al. (2013)].

Břízová et al. (2013) conducted chemical and statistical analysis of the volatile pheromone components produced by males from seven populations of *Anastrepha fraterculus*. Six populations were from three distinct geographic regions of Brazil: Pelotas (Rio Grande do Sul), Vacaria (Rio Grande do Sul), Bento Gonçalves (Rio Grande do Sul) and São Joaquim (Santa Catarina) (south of Brazil); Piracicaba (São Paulo) (southeast of Brazil) and Alagoas (Alagoas) (northeast of Brazil), and the seventh population was from Argentina (Tucumán). In this study, 14 volatile compounds emitted by males including terpenoids, alcohols and aldehydes were identified (Table 1). Multivariate statistical analyses showed that the populations from Vacaria, Pelotas, Alagoas and Tucumán are most dissimilar from the remaining populations (São Joaquim, Bento Gonçalves and Piracicaba) in terms of volatiles produced (Figure 4). The authors hypothesized that there may also be other compounds in the male pheromone mixture that are responsible for the attraction and/or repulsion of conspecific and/or heterospecific females. The differences among the pheromone mixtures released by the males of different Brazilian and Argentinean populations of *Anastrepha fraterculus* might also be regulated by various genes, as previously reported for *Drosophila* spp. (Ferveur et al. 1997, Ferveur 2005). The variability in the male-borne volatile profiles may directly influence the responses of females from these populations and change the manner in which they respond to the pheromone mixtures released by homospecific and heterospecific males (Břízová et al. 2013).

A recent study on chemical and electrophysiological analyses and behavioural bioassays was performed using a population of *Anastrepha fratercul*us from Alagoas (*Anastrepha* sp.3, Alagoas, Brazil) revealing the presence of 29 compounds in headspace samples of *Anastrepha fraterculus* males (Milet-Pinheiro et al. 2015). However, only six compounds, i.e. α-pinene, limonene, (*Z*)-3-nonen-1-ol, (*E*, *Z*)-3,6-nonadien-1-ol, α-farnesene and (*S*, *S*)-(-)-epianastrephin, triggered antennal depolarization in conspecific females. Results from laboratory bioassays showed that synthetic compounds tested individually elicited more behavioral responses than a hexane control, but only the synthetic mixture composed of all EAD-active compounds triggered behavioral response in females similar to the headspace samples of conspecific males. In semi-field conditions, the synthetic mixture was more attractive to females than a hexane control and equally attractive to headspace extracts of males.

**Table 1.** Chemicals identified in the male sex pheromone mixture of *Anastrepha fraterculus* Brazilian morphotypes: *Anastrepha* sp.1 PEL – Pelotas (RS, BR), BEN – Bento Gonçalves (RS, BR), VAC – Vacaria (RS, BR), SAO – São Joaquim (SC, BR), TUC – Tucumán (AR); *Anastrepha* sp.3. AL – Alagoas (AL, BR) [modified after Břízová et al. (2013)].

No.	Compound	*RI*	*Anastrepha* sp.1 PEL	*Anastrepha* sp.1 BEN	*Anastrepha* sp.1 VAC	*Anastrepha* sp.1 SAO	*Anastrepha* sp.1 TUC	*Anastrepha* sp. PIR	*Anastrepha* sp.3 AL
1	*p*-Cymene	1022	++++	+	++	++	++	+	+++
2	2-Ethylhexan-1-ol	1029	+	+++	++++	+	++	+	tr
3	Limonene^†^	1041	++++	++	+++	++++	+++	+	++++
4	(*Z*)-β-Ocimene	1050	++	+	tr	++	+++	+++	-
5	Nonanal	1107	+	++	++++	++	tr	+	+
6	(*Z*)-3-Nonen-1-ol^†^	1159	tr	++	+	+	++	+	+
7	(*E*, *Z*)-3,6-Nonadien-1-ol^†^	1161	tr	++++	+	+++	+++	++++	++++
8	Decenal	1210	++	+	++	+	tr	+	+
9	(*Z*, *E*)-α-Farnesene^†^	1495	+	+	+	++	+	+	+
10	Germacrene D	1498	+	+	+	+	tr	+	+
11	(*E*, *E*)-α-Suspensolide	1506	++	++	+	++	++	++	++
12	(*E*, *E*)-α-Farnesene^†^	1512	++	++	+	++++	++++	++++	+
13	Anastrephin	1617	+	+	+	+	+	+	+
14	Epianastrephin^†^	1621	++	++	+	+++	+	++	+

tr ≤ 0.1 %; + ≤ 3 %; ++ ≤ 10 %; +++ ≤ 20 %; ++++ > 20 %; ^†^Male-borne attractants identified by Milet-Pinheiro et al. (2015) in AL population.

Further comparison of the male-borne chemical profiles of seven populations of *Anastrepha* sp.1 [Bento Gonçalves (Rio Grande do Sul), São Joaquim (Santa Catarina), Pelotas (Rio Grande do Sul)], *Anastrepha* sp.3 (Alagoas), and Andean (Duitama, Ibague, Sibundoy) morphotypes confirmed the previous findings on variability among male pheromone composition (Vaníčková et al. 2015). Male-specific compounds, which were proved by Milet-Pinheiro et al. (2015) to be attractive for *Anastrepha fraterculus* conspecific females, differed qualitatively among the seven studied populations (Vaníčková et al. 2015). The three Andean morphotype populations formed one separated cluster, whereas the one *Anastrepha* sp.3 together with three *Anastrepha* sp.1 populations grouped in a second cluster.

Cladera et al. (2014) stated that the key pheromone components of courtship and their roles as attractants to the two sexes have been overlooked in *Anastrepha fraterculus*. In contrary, here we present evidence proving that the pheromone and its role in the *Anastrepha fraterculus* chemical communication have been the main focus of studies performed by a few groups on the last 14 years. Together, the studies conducted on *Anastrepha fraterculus* pheromones suggest sexual specificity in the production and perception of individual pheromone components in this species. The semi-field bioassays conducted by Milet-Pinheiro et al. (2015) showed that the synthetic mixture of the *Anastrepha fraterculus* male-borne pheromone was effective in attraction of conspecific females, suggesting the use of this mixture for the control of this pest in infested orchards. Therefore, further experiments to evaluate the potential of traps baited with the pheromone mixture are necessary to confirm this hypothesis.

### Cuticular hydrocarbons

Although long range attractants (sex pheromones) are essential for male and female flies to find each other, other substances, including cuticular hydrocarbons (CHs), may function as short range attractants and/or agents which trigger physiological changes, such as induction of receptivity in females and other behaviors associated with mating (Lockey 1991, Howard and Blomquist 2005). Although the primary role of CHs is to protect the insect body against desiccation; secondarily they play an important role in intra- and interspecific communication (Howard and Blomquist 2005, Blomquist and Bagnères 2010). In addition, the sex-specificity in CH profiles indicates the role of CHs in sexual communication (Blomquist and Bagnères 2010). CHs may also serve as species-specific fingerprints useful for delimitation of the putative species hidden within cryptic species complexes (Kather and Martin 2012).

Study of sex- and age-dependent differences in CHs production has been conducted for a laboratory population of *Anastrepha fratercul*us (*Anastrepha* sp.1, Tucumán, Argentina). It was found that sexually mature males had specific unsaturated hydrocarbons (7-monoenes) on their cuticles, which are absent in females (Vaníčková 2012, Vaníčková et al. 2012). The presence of the male specific CHs indicates that these compounds may play a role in the later phase of the mating, when the female touches the male with its front legs and proboscis (Vaníčková 2012, Vaníčková et al. 2012, 2014a, in press). Nevertheless, the exact role of those compounds in pheromone communication of *Anastrepha fraterculus* needs to be further explored.

Vaníčková et al. (2014b, in press) conducted a study including one south and one southeast Brazilian populations [Vacaria (Rio Grande do Sul), Piracicaba (São Paulo), respectively] and compared their chemical CH profiles with flies originated from Peru (La Molina), Mexico (Xalapa), Argentina (Tucumán) and Colombia (Ibague), representing the Peruvian, Mexican, *Anastrepha* sp.1 and Andean morphotypes of *Af* complex, respectively. The authors suggested eight potential taxonomic markers, specifically *n*-hexadecane, *n*-octadecane, *n*-eicosane, 4-methylhexacosane, 7-heneicosene, 7-tricosene, 11-hentriacontene and 7,18-pentatriacontadiene, which could be used for the potential delimitation of males of the particular morphotypes within the *Af* complex. For example, Peruvian and *Anastrepha* sp.1 (Vacaria, Tucumán) morphotypes have unique CH profiles, suggesting CHs could be used to distinguish between these two subspecies.

Nevertheless, when compared the chemical profiles of males and females CHs from *Anastrepha* sp.1 [Bento Gonçalves (Rio Grande do Sul), Pelotas (Rio Grande do Sul), São Joaquim (Santa Catarina)], *Anastrepha* sp.3 (Alagoas) and Andean morphotype (Duitama, Cachipay, Sibundoy), CHs were found to have a limited use for distinguishing between *Anastrepha* sp.1 and *Anastrepha* sp.3 (Vaníčková et al. 2015). In all, the seven *Anastrepha fraterculus* populations analyzed formed two main clusters presenting Andean and Brazilian entities. The *Anastrepha* sp.1 and *Anastrepha* sp.3 populations created monophyletic cluster. These results point out that the relationships between the CH profiles and geographical isolation and/or influence of diet, host fruit, laboratory rearing and possible genetic variability are very complex and not yet understood among the *Af* complex. Therefore, it is necessary to conduct future studies, which will elucidate these complicated relationships of the CH chemical profiles and evaluate their use as specific taxonomic markers. The Brazilian-2 morphotype (*Anastrepha* sp.2) should be also examined for the CH composition and compared with the *Anastrepha* sp.1 and *Anastrepha* sp.3 morphotypes.

## Remarks and conclusions

To date, three sibling species have been documented from the *Af* complex in Brazil. This complex work has relied on a number of analytical methods, such as differences in karyotypes (Mendes 1958, Bush 1962, Solferini and Morgante 1987, Selivon et al. 1996, 2004, 2005a,b), isozymic patterns (Morgante et al. 1980, Steck 1991, Selivon et al. 2005a), and DNA restriction patterns (Santos 1994, Steck and Sheppard 1993, Smith-Caldas et al. 2001, Rocha and Selivon 2004). Egg morphology (Selivon and Perondini 1998, Selivon et al. 2004, Selivon and Perondini 2007) and comparative morphometry of adults (Selivon et al. 2005a, Hernández-Ortiz et al. 2004, 2012, Araujo and Zucchi 2006) have also provided important clues to recognize cryptic species within the complex. Finally, factors involved in reproductive isolation among Brazilian populations, such as courtship behavior, pheromones, and CHs also play a role in distinguishing groups in the complex (Selivon et al. 1999, Santos et al. 2001, Břízová et al 2013, Vaníčková et al. 2014b, in press).

The three Brazilian species exhibit conspicuous differences in the sex chromosomes, both in terms of size and the amount and arrangement of heterochromatic blocks (Selivon and Perondini 2007). They may be also recognized by morphological analysis of the chorion of eggs (Selivon and Perondini 1998, Selivon et al. 2004) and by multivariate morphometrics based on certain linear measurements of the aculeus, wing and mesonotum (Hernández-Ortiz et al. 2012). Morphological data reported in this work show Brazilian morphs are more similar to each other than the other morphotypes described from the Neotropics. A crucial issue was that diverse samples tested by morphometrics corresponded to same populations identified as *Anastrepha* sp.1, *Anastrepha* sp.2 and *Anastrepha* sp.3 by Selivon et al. (2004, 2005a,b). Therefore, the morphological divergence of these samples would be correlated with differences in egg-shell morphology, genetics or reproductive isolation. Further studies need to be conducted involving a wide range of populations of all three Brazilian morphotypes, in order to determine whether the different entities are consistent throughout their geographic and host range.

Out of the eight morphotypes currently recognized within the *Af* complex, there is compelling evidence that Brazilian morphs are the only ones to occur in sympatry in certain regions of the country. For instance, the presence of two karyotypes described from Itaquera (São Paulo) by Solferini and Morgante (1987), and the extreme allozyme variation found in samples of this particular locality by Steck (1991), suggests the coexistence of cryptic species. Nevertheless, a comprehensive study of the distribution of morphotypes throughout Brazil is required to improve the knowledge related to its entire distribution, sympatric areas, to understand the mechanisms of isolation and taxonomic relationships.

Studies on sexual compatibility, acoustic communication, chemical analyses of pheromones and cuticular hydrocarbons are, to some extent, complementary, as the Brazilian populations of the *Anastrepha* sp.1 morph used in these studies were the same. Specifically, *Anastrepha* sp.1 presented by Bento Gonçalves (Rio Grande do Sul), Vacaria (Rio Grande do Sul), São Joaquim (Santa Catarina) and Piracicaba (São Paulo) populations, revealed significant (P < 0.01) differences in the male pheromone composition (Břízová et al. 2013, Table 1), whereas CH profiles of Vacaria and Piracicaba (Vaníčková et al. 2014b, in press), and Bento Gonçalves with São Joaquim (Vaníčková el al. 2015) were comparably similar. Except for the Piracicaba population, all the three populations were sexually compatible between each other (Dias 2012, Joachim-Bravo et al. 2013) and non-significant differences were found between the sounds emitted by the males from these four populations. The chemical profiles and mating compatibility studies were also performed between populations of Pelotas (Rio Grande do Sul) Bento Gonçalves (Rio Grande do Sul), Vacaria (Rio Grande do Sul), and São Joaquim (Santa Catarina) (Dias 2012, Břízová et al. 2013, Joachim-Bravo et al. 2013, Vaníčková el al. 2015).

Together, all the evidence reviewed on three Brazilian entities regarding visual incompatibility, acoustic communication and chemical profiles suggest that the combination of all three types of signals will be necessary for the development of an effective pest monitoring and management program since these studies pointed out that (i) sexual specificity in the production and perception of individual pheromone components in this species might exist (ii) synthetic mixture of the *Anastrepha fraterculus* male-borne pheromone was effective in attraction of conspecific females, suggesting the use of this mixture for the control of this pest in infested orchards (Milet-Pinheiro et al. 2015), (iii) acoustic signals can be used as lures to attract tephritid flies (Webb et al. 1983, Mankin et al. 2004, Mizrach et al. 2005), and (iv) the CH profiles are species- and sex-specific, suggesting their use as possible chemotaxonomic markers for *Af* complex delimitation.

Future studies focused on electrophysiological and behavioral studies of the chemical communication of *Af* complex could help to understand the complex relationships between the three Brazilian entities. Research on mating behavioral sequences of the three Brazilian entities of the *Af* complex as well as basic knowledge about *Anastrepha fraterculus* sexual communication could help to unravel mate assessment and mate choice dynamics, leading to the development of behavior-based control strategies and novel control tools for integrated pest management programs (Benelli et al. 2014a). Complementary molecular, genetics and morphological studies should be performed using the identical populations, in order to allow comparisons of all data obtained and subsequent implementation of effective control strategies of these pests.

From an applied perspective, area-wide integrated pest management programs based on the Sterile Insect Technique (SIT) cannot use only one *Anastrepha fraterculus* population to cover all Brazil. However, one population may be used in SIT programs covering wide areas that share the same morphotype, male courtship behavior and same time of the day when matings occur (Cladera et al. 2014). The use of lured semiochemicals (sex pheromones together with host fruit kairomones) combined with the SIT could improve the integrated pest management program by reducing dispersal, longevity and fecundity of *Anastrepha fraterculus* adults from the same morphotype. Despite an increasing number of studies demonstrating reproductive compatibility in the *Anastrepha* sp.1 morphotype and the partial incompatibility between *Anastrepha* sp.1 and *Anastrepha* sp.2, much remains to be learned about the *Anastrepha* sp.2, and *Anastrepha* sp.3 morphotypes.
